# The hidden factors in impact factors: a perspective from Brazilian science

**DOI:** 10.3389/fgene.2013.00130

**Published:** 2013-07-11

**Authors:** Renata C. Ferreira, Fernando Antoneli, Marcelo R. S. Briones

**Affiliations:** Laboratório de Genômica Evolutiva e Biocomplexidade, Departamento de Microbiologia, Imunologia e Parasitologia, Universidade Federal de São PauloSão Paulo, Brazil

Contrary to all current international recommendations on evaluation of academic achievement the evaluation of graduate programs in Brazil relies heavily on journal impact factors (Garfield, 2006; San Francisco Declaration on Research Assessment, 2013). The governmental agency CAPES from the Education Ministry monopolize this evaluation and pressure programs by the distribution of funding resources and departmental fellowships conditioned to adherence to a journal classification system called “Qualis” which is a discretization of the continuous distribution of journals ranking by their impact factors (Greenwood, 2007). In several institutions the graduate committee authorizes professors to act as thesis advisors only if in a certain period (e.g., 4 years) they publish at least one paper in a journal classified as “Qualis A2.” This classification has seven categories with decreasing impact factor ranges (A1, A2, B1, B2, B3, B4, B5, and C, http://www.capes.gov.br/avaliacao/qualis) and has certain percentile adjustments depending on the field of research. This has been hailed as a major cause for the enhancement of Brazilian scientific output although this system has several critics, demanding a profound review of evaluation criteria, and a proper adaptation to international guidelines (Rocha-e-Silva, 2009; Hermes-Lima, 2013). Most critics suggest that the excessive concern with publication in certain journals is in fact reducing the originality. Recently, to dismay of CAPES, a survey of the Brazilian science publication output, from 2001 to 2011, has shown that although Brazil has climbed from 17th place to 13th place in the total number of papers it has dropped from 31th to 40th place in citations (Rughetti, 2013).

To investigate the “Qualis” effect we obtained journal citation data from Thomson Reuters for six journals in the Biological area and calculated, from raw data, the impact factors (mean), standard deviations, median, and mode. The journals selected are in the CAPES classification “Biology III” and correspond to the highest impact factor of A2 and B1, the middle impact factor of A2 and B1 and the lowest impact factors of A2 and B1. The A2 category includes 180 journals and B1 includes 255 journals.

**Figure 1:**
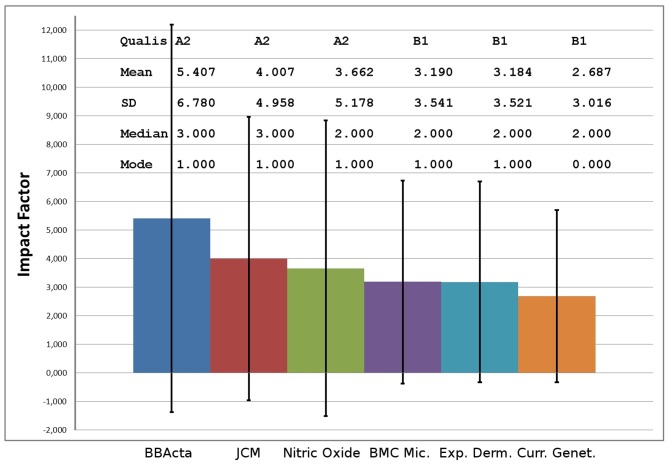
**Statistics of impact factors of journals indexed in the Brazilian Qualis.** BBActa, Biochemical and Biophysical Acta; JCM, Journal of Clinical Microbiology; BMC Mic., BMC Microbiology; Exp. Derm., Experimental Dermatology and Curr. Genet., Current Genetics; SD, Standard Deviation of the impact factor (error bars).

These journals lie at the “exclusion zone,” in other words the frontier between A2 and B1 exactly where a professor may or may not be promoted to formal advisor status. As expected, the distributions of citations for these six journals follow power laws (Baum, 2011). As widely known power laws cannot be compared on the basis of their means (the impact factor) but preferably on their medians. Also, the standard deviations are larger than the means, showing the over-dispersed nature of the data and how the mean is meaningless in this case. In Figure 1 the journal ranking is depicted showing that the differences between journal means (impact factors) are significantly smaller than the standard deviations. This shows that the “Qualis” categories and journals considered are not statistically different. How can scientists and publications from graduate programs be compared on the basis of these measurements? Could this be, at least in part, responsible for the drop in citations of Brazilian research? As shown by others (Editorial, 2005) is this an effect of free-riding on power law outliers?

This shows yet another nationwide example of misuse of journal based metrics to evaluate individual scientists and its catastrophic consequences when, contrary to recommended by the International Mathematical Union (IMU Report, 2011), it is imposed by governmental agencies. In a country that is desperately trying to move from a peripheral scientific and technological condition to be among the major players, the journal impact factor based “Qualis” might be bad news.
